# Ten Minutes of Core Stabilisation Exercise Result in Local Exercise‐Induced Hypoalgesia in Patients With Chronic Unspecific Low Back Pain

**DOI:** 10.1002/ejp.4794

**Published:** 2025-02-08

**Authors:** Fabian Tomschi, Andre Zschunke, Thomas Hilberg

**Affiliations:** ^1^ Department of Sports Medicine University of Wuppertal Wuppertal Germany

## Abstract

**Background:**

Core stabilisation training is known to be effective in managing pain in patients suffering from chronic low back pain (CLBP). Yet, acute effects of core stabilisation exercise on exercise‐induced hypoalgesia (EIH) are largely unknown. This study aimed to examine the EIH effects of an easy‐to‐perform core stabilisation exercise in CLBP patients and to explore associations between EIH and potential influencing factors (i.e., physical activity, catastrophizing, kinesiophobia, subjective pain state and exercise exertion).

**Methods:**

Thirty patients with unspecific CLBP finished this randomised controlled crossover trial. Patients performed a 10‐min isometric core stabilisation exercise and a 10‐min control session. Before and after, pain sensitivity was measured via pressure pain thresholds [Newton/cm^2^] locally (low back; PPT_local_) and remotely (forehead, thumb; PPT_remote_). Correlation analyses were performed between EIH and influencing factors.

**Results:**

A ‘Time’ × ‘Intervention’ interaction (*p* < 0.001) was observed for PPT_local_ with post hoc analysis revealing higher values post exercise (*p* < 0.001; pre: 56.6 ± 20.6, post: 67.5 ± 26.1). No differences were observed for the control session (*p* = 0.894; pre: 58.5 ± 24.0, post: 58.4 ± 23.3). No such effect was observed for PPT_remote_ (*p* = 0.014). Post hoc analyses showed no differences following the exercise session (*p* = 0.103; pre: 41.3 ± 12.5, post: 42.5 ± 13.6), while lower PPT_remote_ post values were observed post control compared to pre values (*p* = 0.031; 42.5 ± 14.5, post: 41.3 ± 13.7). The only significant moderate correlation was observed between ΔPPT_local_ of the exercise session and catastrophizing with *rho* = −0.381.

**Conclusion:**

A 10‐min isometric core stabilisation exercise results in local lumbar EIH, while no systemic effects are observed. A higher degree of catastrophizing is associated with lower hypoalgesic responses.

**Significance:**

This study shows for the first time that a brief and easy‐to‐perform 10‐min core stabilisation exercise produces significant local pain relief (EIH) in patients with unspecific CLBP. The effect is localised to the lumbar region, with no observed impact on remote sites. Higher pain catastrophizing seems to be linked to reduced hypoalgesic response. These findings support the use of short core stabilisation exercises as an effective, immediate, non‐pharmacological pain management strategy for these patients.

## 1 Introduction

Exercise‐induced hypoalgesia (EIH) is a well‐documented phenomenon in which a single session of exercise leads to a transient reduction in pain sensitivity (Naugle et al. 2012). This physiological response, characterised by an acute increase in pain thresholds as well as decrease in pain perception (e.g., pain sensitivity), has been observed consistently across the healthy population (Vaegter and Jones 2020). Ample evidence exists showing that both aerobic and resistance exercises can trigger EIH via modulation of central and peripheral pain pathways, likely through mechanisms such as the activation of endogenous opioid and endocannabinoid systems, increased tissue perfusion and enhanced descending pain inhibitory control (Tomschi, Schmidt, et al. 2024; Wewege and Jones 2021).

However, research dealing with EIH in pain patients shows equivocal results with varying degrees of EIH responses among different chronic pain populations, suggesting a complex interaction between the type of pain, its chronicity, and the exercise modality used (Naugle et al. 2012; Rice et al. 2019). Most likely, several factors contribute to the inconsistency in EIH responses observed in chronic pain populations. These include, among others, adaptations in the central nervous system to chronic pain, such as central sensitisation (Latremoliere and Woolf 2009), and alterations in endogenous descending pain inhibiting pathways (Sluka et al. 2018) that might alter pain processing and might result in varying levels of pain sensitivity (hyperalgesia, hypersensitivity or allodynia) (Latremoliere and Woolf 2009). Besides, psychosocial factors (Munneke et al. 2020) as well as further disease specific factors most likely contribute to this inconsistency.

The effectiveness of EIH in patients with chronic unspecific low back pain (CLBP) is disputed and existing literature reveals heterogeneous results as well as a variety of exercise regimens being used. Some studies show that exercise can result in hypoalgesia in this patient group (Hoffman et al. 2005; Meeus et al. 2010; Paungmali et al. 2017; Pinho et al. 2023), while others show that exercise can also result in no change of pain perception (Kuithan et al. 2019; Pinho et al. 2023; Santos et al. 2022; Sitges et al. 2021), or in some cases even hyperalgesia (i.e., increase of pain sensitivity) (Vaegter et al. 2021).

Core stabilisation exercises can be defined as exercises that strengthen and enhance coordination of the group of trunk muscles surrounding the spine, primarily through the co‐activation of the transversus abdominis, quadratus lumborum and lumbar multifidus (Martuscello et al. 2013), along with contributions from, among others, internal and external obliques, rectus abdominis, the pelvic floor, iliac psoas and diaphragm. Core stabilisation exercises have been promoted as an effective method for managing CLBP (Akuthota et al. 2008; Hlaing et al. 2021; Oliva‐Lozano and Muyor 2020; Shinkle et al. 2012) by means of maintaining and improving neuromuscular control as well as postural and spinal stability (Hlaing et al. 2021) and are therefore recommended for patients (Owen et al. 2020; Searle et al. 2015). Even though this training method has been shown to be highly effective in multi‐week intervention studies (Fernández‐Rodríguez et al. 2022; Hlaing et al. 2021; Owen et al. 2020), to best of our knowledge, no study explored the acute effects of core stabilisation exercises using the own body weight on EIH.

Based on these considerations this study aims investigate the acute effects of a short (10 min) and easy to perform (no additional weights or machines needed) core stabilisation exercise using the own body weight on EIH. Specifically it is aimed (1) to explore whether this exercise results in EIH in CLBP patients compared to a control session; (2) to explore whether these effects differ between local and remote measurement sites; and (3) to identify potential relationships between EIH and influencing factors, such as pain status, kinesiophobia, pain catastrophizing, physical activity status, and perceived subjective exertion during exercise. It is hypothesized that (1) core stabilisation exercises will result in EIH in CLBP patients, with a reduction in pain sensitivity compared to a control session, (2) the magnitude of EIH will be higher at local (lumbar back) landmarks than at remote landmarks, and (3) EIH outcomes will be influenced by the above mentioned influencing factors.

## 2 Methods

### 2.1 General Study Design

This study was performed in a randomised controlled crossover design consisting of three study visits. In the first visit, patients were screened for eligibility and the study procedure was explained. After patients provided written consent for participation, a familiarisation of pressure pain thresholds (PPT) measurements was performed and the core exercise was explained and practiced. The next two visits consisted of an exercise and control session (see below for more details). Patients were randomly assigned to one of two sequence groups using opaque sealed envelopes, which patients draw by themselves from a container. One group initially performed core exercise, followed by the rest intervention 1 week later, while the other participated in the opposite sequence. The washout phase of 1 week was included to ensure that the participants were not influenced by the interventions (carry‐over effect). During the visits for the exercise and control session, pre PPT measurements (see below for more details) were conducted after the participants had rested for 10 min. Immediately, following these measurements, the exercise/control session was performed. Immediately post (approximately 60 s post termination), PPT measurements were again performed. Patients were asked not to engage in any strength exercise or any similarly intensive physical activity 48 h before any measurement (Santos et al. 2022). Additionally, patients were instructed to refrain from taking pain medication 24 h before the intervention. The study and the used protocols were approved by the local ethics committee. These protocols are in line with the Declaration of Helsinki. Participants gave written informed consent to participate in the study.

### 2.2 Participants

Using G‐Power (Version 3.1.9.4) and following Santos et al. (2022), a medium effect size of *f* = 0.3 was estimated. With a significance level (alpha) of 0.05 and a statistical power of 0.8, a minimum of 24 participants (12 per group) were needed for this crossover design. Considering an anticipated dropout rate of 30%, 32 patients were aimed to be included in this study.

In this study, the participants consisted of patients between the ages of 18 and 65 suffering from diagnosed unspecific CLBP (Kuithan et al. 2019). Patients were included if they experienced lower back pain between the twelfth thoracic vertebra (Th12) and the first sacral vertebra (S1) with no specific identifiable cause. The subjective average lower back pain intensity during the last 12 weeks had to be at least three on the Numerical Rating Scale (NRS) ranging from 0 (no pain) to 10 (worst imaginable pain) (Kuithan et al. 2019; Paungmali et al. 2017; Santos et al. 2022).

Exclusion criteria included specific causes of back pain syndromes in the examined area or those that could potentially interfere with the course of the study, such as for instance intervertebral disc herniation, spinal stenosis, spondylolisthesis, or ankylosing spondylitis. A history of spinal fractures as well as radiating leg pain (pain, numbness, or tingling extending into the lower limbs below the buttock fold), were also exclusion criteria, as were simultaneous systemic rheumatic or neurological diseases (Kuithan et al. 2019; Paungmali et al. 2017). Pregnancy, surgical treatment for lower back pain, spinal conditions requiring surgical intervention and patients with psychiatric, motor, or cognitive deficits, or communication disorders that prevented participation in the study, were also excluded. Potential patients were screened for inclusion from a local health centre in Germany using a self‐established questionnaire as well as the German Pain Questionnaire (Nagel et al. 2002) that allowed for determining eligibility and exclusion criteria. The study was conducted between the 27 of June 2023 and 14 of October 2023.

### 2.3 Pressure Pain Thresholds

To assess pain sensitivity and EIH, mechanical PPT were employed using the digital FPX 25 Compact algometre (Wagner Instruments) and participants were informed about the procedure of the PPT measurement with instructions given in a standardised manner in every visit. The algometer's one cm^2^ rubber‐tipped probe was used to apply pressure to the measurement points, with a linear increase of ten Newton (N) per second. Patients were instructed to say ‘stop’ when they first perceived the stimulus as painful. The measurement was then terminated and the force peak value was recorded in N per cm^2^. The testing sites were located locally at the lower back and at two remote landmarks conducted in a randomised order. Local measuring points were at the lower back with four measurement points on each side of the spine, each two centimetres away from the spine between the twelfth thoracic vertebra and the first sacral vertebra at the level of the musculus erector spinae. The vertical distance between each measurement point was 2.5 cm and measurements were performed directly on the muscle belly of the erector spinae at the level of the lumbar vertebrae L 1/2, L 2/3, L 3/4, and L 4/5 as done before (Kuithan et al. 2019). In addition, measurements were conducted at two remote (non‐exercise‐related landmarks) on the dominant thenar eminence (thumb pad) and the forehead (Gajsar et al. 2017; Tomschi et al. 2023). Each measurement was performed twice with a 10‐s break between measurements. The mean of two consecutive measurements at each point was used for analyses (Balaguier et al. 2016). Only when deviations exceeded 10 N were observed a third measurement was conducted. A threshold of 140 N was predetermined in order to avoid tissue damage. If participants did not report pain below this threshold, a PPT value of 140 N was recorded (Tomschi, Herzig, and Hilberg 2024). PPT measurements were performed by one examiner to avoid any inter‐rater discrepancies.

### 2.4 Exercise Intervention

**FIGURE 1:**
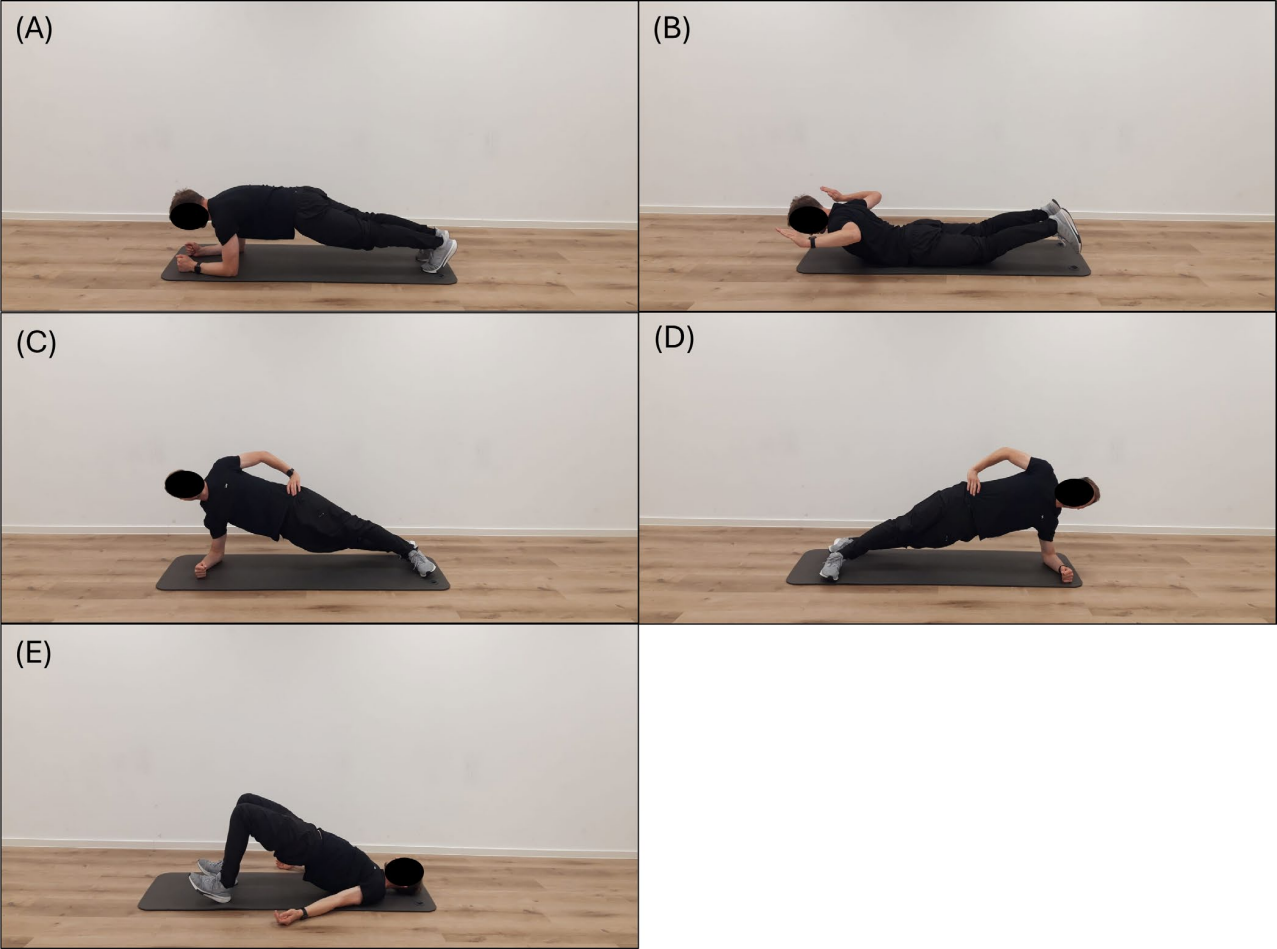
Pictures of the five exercises performed during the 10‐min core stabilisation training: (A) forearm plank, (B) static swimmers, (C) right‐side plank, (D) left‐side plank, and (E) supine bridge. Each exercise was performed three times in this order for 30 s followed by 10 s rest resulting in a total training duration of 10 min.

The exercise intervention consisted of isometric strength exercises, including forearm plank, static swimmers, right‐side plank, left‐side plank, and supine bridge (Figure 1) and were performed in this order. Each of the mentioned exercises was held for 30 s, followed by a 10‐s rest period. Three consecutive rounds of exercises were completed, resulting in a total exercise duration of 10 min. Participants were instructed to perform the exercises as standardised as possible and throughout the entire duration of the load. To assess the intensity of individual exercises and the overall exercise program, participants were asked to rate the perceived exertion (RPE) using the Borg Scale (6 = no exertion at all, 20 = maximum exertion) just at the end of each exercise. After completing the entire exercise session, participants were also asked to provide feedback on how strenuous they perceived the overall exercise to be also using the Borg Scale.

### 2.5 Control Intervention

A quiet rest of 10 min was used as the control intervention. Patients were in a seated position in a secluded and quiet room. Pre and post, PPT measurements were conducted.

### 2.6 Questionnaires

The seven‐item International Physical Activity Questionnaire—Short Form (IPAQ) was used to assess the total physical activity (MET‐h/week) and sedentary time (h/day) (Craig et al. 2003). The 11‐item Tampa Scale for Kinesiophobia (TSK) was used to measure the level of fear of movement or physical activity due to potential pain or injury (Rusu et al. 2014). The 13‐item Pain Catastrophizing Scale (PCS) was used to assess the patients' thoughts and feelings they may experience when having pain (Meyer et al. 2008). For analyses, the total scores of the IPAQ, TSK and PCS were used.

### 2.7 Statistics

The data were analysed using the statistical software IBM SPSS Statistics 29.0 for Windows. A statistical analysis was conducted on all PPT measurements taken at the 10 body sites individually. Further, values were combined into one local value (PPT_local_) where the mean value was established for the measurement points at the lower back and into one remote value (PPT_remote_) with the mean value for the distal measurement points (forehead and thumb pad) as done before (Pinho et al. 2023). The Shapiro–Wilk test was used to confirm normal distribution. The significance level for all tests was set at *p* ≤ 0.05.

The main analysis was conducted to test the 1st research question. A two‐way repeated‐measures ANOVA was used for PPT_local_ and PPT_remote_ with the factors ‘Time’ (pre, post) and ‘Intervention’ (Control, Exercise). This analysis was also conducted for each individual measurement point. In the case of a significant main or interaction effect, subsequent Bonferroni adjusted post hoc tests were calculated. Effect sizes are presented as partial eta‐squared (*η* ^*2*^_*partial*_) with values of 0.01 representing a small, 0.06 a medium, and ≥ 0.14 a large effect, respectively (Cohen 1988).

To test the second research question, ΔPPT values were calculated by subtracting the pre values from the post values for both session. A two‐way repeated‐measures ANOVA with the factors ‘Measurement site’ (PPT_local_, PPT_remote_) and ‘Intervention’ (Control, Exercise) was calculated.

In the light of the third research question, Spearman correlation analyses using rho (*ρ*) were calculated to assess the relationships between ΔPPT_local_ and ΔPPT_remote_ of the exercise session and potential influencing factors that is, acute (NRS) and chronic pain status (NRS of the last 12 weeks), Body‐Mass‐Index, kinesiophobia (TSK), pain catastrophizing (PCS), physical activity status (IPAQ), and perceived subjective exertion during exercise (RPE). Correlation coefficients are presented as rho and can be interpreted with rho = 0.10, rho = 0.30, and rho = 0.50 representing a weak, moderate and large correlation, respectively (Cohen 1988).

To account for any carryover effects, PPT pre‐ values of each body site were compared between the two sessions using paired *t*‐tests. Data are presented as mean ± standard deviation unless otherwise specified.

## 3 Results

Thirty‐two participants were included in this study. Two participants dropped out due to personal reasons (*n* = 1) and due to a too uncomfortable feeling of the PPT measurements (*n* = 1). These two data sets were not considered in any analyses. Patients' anthropometric and disease specific data are presented in Table 1. No adverse effects (such as for instance nausea, dizziness, or pain flare ups) were observed throughout the study.

**TABLE 1:** Anthropometric and disease specific data of included patients. Data are presented as mean ± standard deviation (range).

Parameter	*N* = 30
Age [years]	42.3 ± 10.5 (27.0–60.0)
Weight [kg]	79.1 ± 15.2 (40.0–108.0)
Height [m]	1.77 ± 0.11 (1.55–2.05)
BMI	25.2 ± 4.4 (13.1–33.4)
Mean NRS last 12 weeks [0–10]	4.2 ± 1.1 (3.0–7.0)
Physical activity time [MET‐h/week]	46.7 ± 30.7 (8.4–117.3)
Sitting time [h/day]	6.0 ± 2.9 (1.5–12.0)
Catastrophising [points]	15.8 ± 7.9 (0.0–36.0)
Kinesiophobia [points]	19.5 ± 4.4 (12.0–29.0)

Abbreviations: BMI = body mass index, MET = metabolic equivalent of task, NRS = numeric rating scale.

**FIGURE 2:**
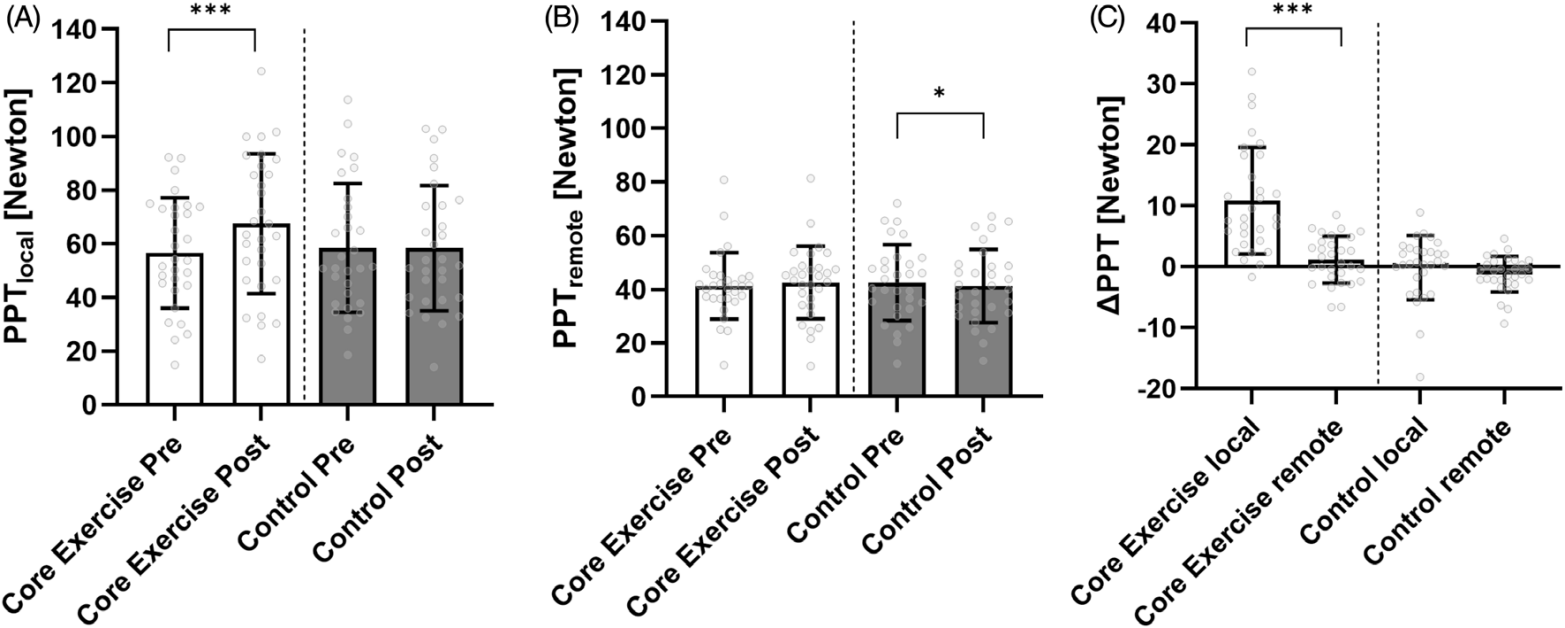
Results of the core training and control session on pressure pain thresholds (PPT) of local (A) and remote (B) measurement sites as well as local and remote ΔPPT values (C) resulting from the two sessions, respectively. Data are presented as mean and standard deviation.

Regarding the first research question, results of the two‐ way ANOVA with PPT_local_ as the dependent variable revealed a significant ‘Time’ × ‘Intervention’ interaction (F(1, 29) = 31.823, *p* < 0.001, *η* ^*2*^_*partial*_ = 0.523; Figure 2a). A main effect was observed for ‘Time’ (F(1, 29) = 36.477, *p* < 0.001, *η* ^*2*^_*partial*_ = 0.557) but not for ‘Intervention’ (F(1, 29) = 3.242, *p* = 0.082, *η* ^*2*^_*partial*_ = 0.101). Post hoc analysis showed higher PPT_local_ values post exercise (*p* < 0.001) with no differences resulted from the control session (*p* = 0.894). Post exercise PPT_local_ values were significantly different between exercise and control (*p* < 0.001). The relative change of PPT_local_ was 18.79% ± 12.64% and 0.01% ± 8.84% for the exercise and control session, respectively. The two‐way ANOVA with PPT_remote_ as the dependent variable revealed a significant ‘Time’ × ‘Intervention’ interaction (*F*(1, 29) = 6.845, *p* = 0.014, *η* ^*2*^_*partial*_ = 0.191; Figure 2b). No main effect was observed for ‘Time’ (F(1, 29) = 0.001, *p* < 0.977, *η* ^*2*^_*partial*_ = 0.000) or ‘Intervention’ (F(1, 29) = 0.000, *p* = 0.995, *η* ^*2*^_*partial*_ = 0.000). Post hoc analyses showed that no pre‐post differences occurred following the exercise session (*p* = 0.103), while lower PPT_remote_ post values were observed after the control session compared to pre values (*p* = 0.031). Post exercise PPT_remote_ values were not different between exercise and control (*p* = 0.434). The relative change of PPT_remote_ was 2.25% ± 9.89% and − 2.26 ± 7.06% for the exercise and control session, respectively. Data as well as results of the statistical analyses including main and interaction effects of each measurement point are to be found in Table S1.

In the light of the second research question, results of the two‐way ANOVA revealed a significant ‘Measurement site’ × ‘Intervention’ interaction (F(1, 29) = 19.725, *p* < 0.001, *η* ^*2*^_*partial*_ = 0.405; Figure 2c). Post hoc analyses revealed that ΔPPT were significantly different between PPT_local_ and PPT_remote_ in the exercise session (*p* < 0.001), while no such differences were observed in the control session (*p* = 0.355).

**FIGURE 3:**
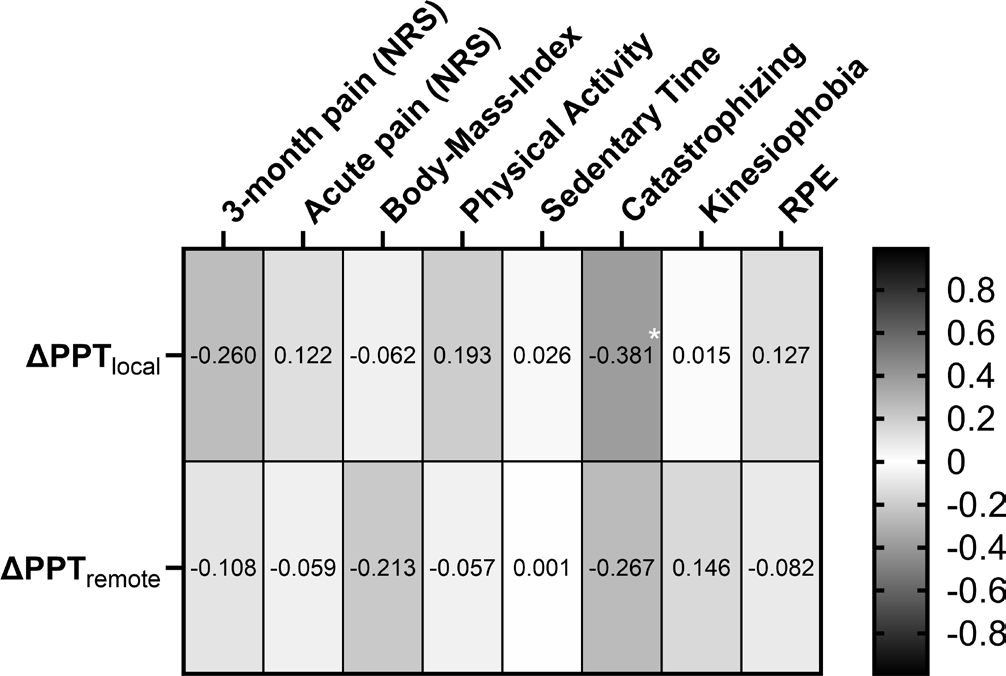
Correlogram of the correlation analyses between ΔPPT of local and remote landmarks and potential influencing factors (i.e., subjective pain states, subjective physical activity behaviour, catastrophizing, kinesiophobia, and rate of perceived exertion (RPE)) during the core training. Results are presented as rho (*ρ*)‐values. * indicates a significant correlation *p* < 0.05. The darker the colour of the boxes the stronger the correlation is (see bar right to the correlogram). NRS = numeric rating scale, PPT = pressure pain threshold, RPE = rate of perceived exertion.

Results of the correlation analyses dealing with the third research question are presented as a correlogram in Figure 3. The only significant moderate correlation is observed between ΔPPT_local_ of the exercise session and pain catastrophizing with *rho* = −0.381 (*p* = 0.038) meaning that a higher degree of catastrophizing is associated with lower hypoalgesic response.

**FIGURE 4:**
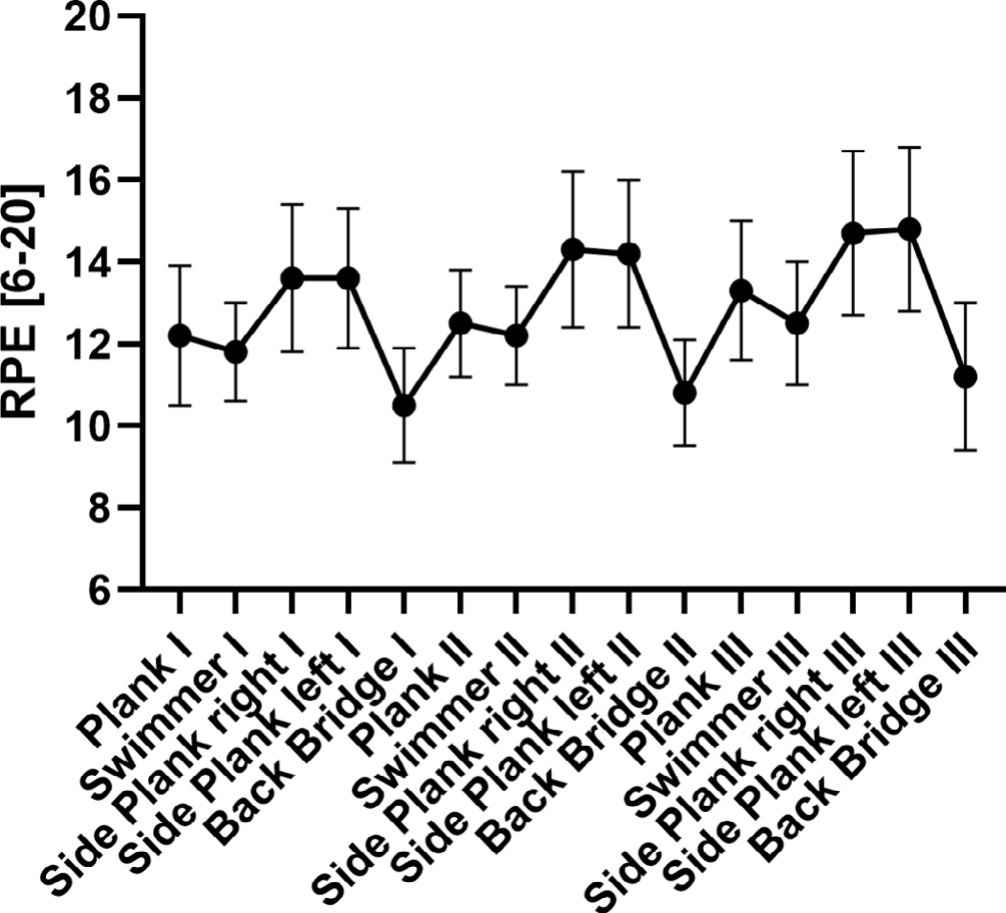
Course of the rate of perceived exertion (RPE) of patients throughout the 10‐min core stabilisation training.

No carryover effects were observed for PPT pre‐ values for local and remote PPT (PPT_local_: *p* = 0.425; PPT_remote_: *p* = 0.455). The entire core exercise was perceived as 13.07 ± 0.98 [median: 13.00, interquartile range: 13.00, 13.25] on the 6–20 RPE Borg scale. The course of the RPE values during the exercise is presented in Figure 4.

## 4 Discussion

This study sought to explore the EIH effects of a 10‐min core stabilisation exercise on EIH in patients suffering from unspecific low back pain. The exercise was designed to be easy to perform by using the own body weight without the need for additional weights or machines and no adverse events occurred. The main results of this research support hypothesis (1) as the exercise resulted in EIH compared to the control session. Further, hypothesis (2) is confirmed since these EIH effects are only observed locally in the lumbar region (L1 to L5) and not remotely. Lastly, results of the correlation analyses on partially support hypothesis (3) as the hypoalgesic effects are solely associated with the patients' pain catastrophizing but not with other potential influencing factors.

More particularly, results presented herein reveal EIH at local measurement sites following the 10‐min core stabilisation exercise in this patient group with significantly higher post PPT values for pooled PPT_local_ and a relative increase of 19%. This was also observed in all eight individual lumbar landmarks (L 1/2 to L 4/5) with relative increases ranging from 14.8% (L 2/3 right) to 24.8% (L 3/4 right; see Table S1). In contrast, no such effects were observed remotely at the hand or forehead (relative change in PPT_remote_: 2%).

The effectiveness of EIH in patients with CLBP has not yet been sufficiently investigated, and existing literature presents heterogeneous findings. Some studies demonstrate that exercise induces hypoalgesia in this patient population (Hoffman et al. 2005; Meeus et al. 2010; Paungmali et al. 2017), whereas others report no changes in pain perception (Kuithan et al. 2019; Pinho et al. 2023; Santos et al. 2022; Sitges et al. 2021), or, in some cases, even observe hyperalgesia (i.e., increased pain sensitivity) following exercise (Vaegter et al. 2021). The heterogeneity of these results as well as the discrepancy to the results presented herein must be primarily be attributed to the use of different exercise modalities across studies. The present study used core stabilisation exercises with a isometric activation of the trunk muscles surrounding the spine including the lower back extensor muscles. These contract and generate tension, which increases the metabolic demand, and result in an increased local blood flow (Paungmali et al. 2016) to deliver the necessary energy resources and remove metabolic by‐products. The other mentioned studies made use of modalities such as lifting exercises (Kuithan et al. 2019), stair‐climbing tasks (Sitges et al. 2021), six‐minute walking tests (Vaegter et al. 2021), ergometer training (Hoffman et al. 2005; Meeus et al. 2010), and functional training (Santos et al. 2022), which are only partially comparable to the present study as these exercises did not focus on trunk muscle activation. One other study also employed core stabilisation exercises (Paungmali et al. 2017), yet, these were different compared to the exercises used in the present study. In this study, patients performed a lumbopelvic core stabilisation exercise (LPCS) protocol. The LPCS involved different exercises (core with alternate hip abduction, core with alternate knee raise, core with both arms adduction, core with both arms extension, core with alternate arm lift, core with alternate leg lift, and core with alternate leg and arm lift), executed on a pilates power gym transformer. Results revealed that the LPCS exercise induced a significant improvement in PPT of about 8% in the lumbar region compared to the placebo (passive cycling) and the control session. The present study induced an PPT increase of 19% in the lumbar region. Of note is that the landmark for PPT measurement was the most painful spot identified by the individual participant (Paungmali et al. 2017). Hence, this study as well as the present study show that core exercises seem to be promising to induce local hypoalgesia.

The mechanisms underlying the EIH phenomenon are still not well understood, beyond the widely accepted notion that EIH is a multicausal phenomenon involving both central and peripheral/local neurophysiological processes (Rice et al. 2019). Systemic central mechanisms are most likely linked to endogenous inhibitory mechanisms at the level of the central nervous system (CNS). Via the activation of the endocrine stress axis, also known as HPA axis (hypothalamic–pituitary–adrenal axis), especially vigorous exercise results in an increase catecholamine production (i.e., adrenaline, noradrenaline) (Hilberg et al. 2003), which in turn results in a production of neuroendocrine hormones such as the endogenous opioid beta‐endorphin or the endocannabinoid anandamide (AEA) (Brooks et al. 1988; Heyman et al. 2012). At the level of the CNS, pain‐inhibiting neurotransmitters (such as endocannabinoids and opioids) lead to acute pain inhibition as a result of exercise. Yet, these CNS mechanisms are thought to be especially activated when a high metabolic demand is present as observed, for instance, in high‐intensity aerobic exercise (Tomschi, Schmidt, et al. 2024) or high intensity functional training, as also observed in CLBP patients (Santos et al. 2022). This might be one explanation for the lack of EIH observed at remote measurement sites (i.e., forehead & hand). The present isometric core exercises were subjectively perceived as “somewhat hard” (RPE of 13.07 ± 0.98) and the low intensity and duration of the task was most likely not vigorous enough to induce large systemic effects, since remote changes are typically seen after excessive or vigorous exercise (Micalos and Arendt‐Nielsen 2016; Naugle, Naugle, Fillingim, Samuels, and Riley, 2014; Tomschi et al. 2022, Tomschi, Herzig, and Hilberg, 2024). Contrastingly, large local EIH effects are observed in the present study. Here, mechanisms close to the exercising muscles probably further contribute (or add further up) to the above mentioned central pain inhibiting mechanisms, resulting in measurable local EIH effects at sites near the exercising muscles (Micalos and Arendt‐Nielsen 2016).

At the level of peripheral muscle tissue, intensive exercise primarily triggers the release of nociceptive substances, such as glutamate, lactate, and pyruvate, leading to acute exercise induced muscle pain (Rosendal et al. 2004). Concurrently, pain inhibition occurs in the peripheral muscle tissue through the peripheral release and uptake of antinociceptive substances, such as peripheral endocannabinoids and opioids (Koltyn et al. 2014; Micalos and Arendt‐Nielsen 2016), which modulate nociceptive primary afferents (Koltyn et al. 2014). These antinociceptive mechanisms outweigh the acute pronociceptive response resulting in local analgesia post exercise. This notion that antinociceptive substances can act peripherally is supported by research indicating reduced EIH in limbs occluded with a cuff during exercise, suggesting that these peripheral antinociceptive substances might be circulating in the bloodstream (Jones et al. 2017). It was previously reported that plasma beta‐endorphin is increased acutely in CLBP patients following a 15 min lumbar core stabilisation training (Paungmali et al. 2018) as well as a 35–40 min functional training consisting of dynamic whole‐body exercises (Santos et al. 2022).

The core‐stabilisation exercise used in this study primarily aims to enhance motor control of the lumbopelvic region, which might be another possible explanation for the local EIH observed. Effective motor control allows precise coordination of the stabilising trunk muscles (Hodges and Richardson 1996; Paungmali et al. 2017). By acutely activating these muscles, they increase intersegmental spinal stiffness, which helps stabilise the lumbar vertebrae and prevents excessive shear forces (Quint et al. 1998). These are common sources of mechanical irritation and nociceptive input in individuals with low back pain (Cholewicki and McGill 1996; Lee and McGill 2017). By reducing these mechanical triggers of nociception, core‐stabilisation exercises likely decrease peripheral nociceptive input, which may contribute to the observed increase in pain thresholds in the lumbar region post exercise.

Regarding the association between EIH and further influencing factors, results indicate that only pain catastrophizing is correlated negatively with EIH by moderate effect sizes. Hence, patients with a higher degree of catastrophizing show a reduced hypoalgesic effect. This observed relationship between pain catastrophizing and EIH has been described in healthy participants before, where greater pain catastrophizing was associated with smaller reductions in temporal summation following 3‐min isometric handgrip exercise performed at 25% of maximum voluntary contraction (Naugle, Naugle, Fillingim, and Riley 2014). Besides, studies in younger adults show that higher pain catastrophizing is associated with less pain inhibition during a conditioned pain modulation test (Weissman‐Fogel et al. 2008). In this context, a recent study demonstrated that a maximal graded exercise cycling test resulted in EIH across local, regional, and global body regions in pain‐free, predominantly female nurses. Regression analyses indicated that pain catastrophizing along with perceived social support were the most significant psychosocial factors, accounting for up to one‐quarter of the variance in EIH (Johnsen et al. 2023). Contrastingly, research in healthy participants also shows a neglectable relationship between these two constructs (Ohlman et al. 2018) and to the best of the authors knowledge, no research previously described this association in CLBP patients. Looking at the other explored factors of the present study that were previously shown to possibly affect EIH, such as subjective pain states (Christensen et al. 2022; Rice et al. 2019), subjective physical activity and sedentary time (Ohlman et al. 2018; Schmitt et al. 2020), or kinesiophobia (Vaegter et al. 2018), results hint to the assumption that these variables, which are relevant in clinical pain management and have previously been linked to EIH effects, are only little associated with EIH responses. Based on the results presented herein, EIH seems to be only altered in the patient's degree of pain catastrophizing.

### 4.1 Strengths and Limitations

The major strength of this study is that a 10 min isometric core stabilisation exercise was established that can be performed anywhere without any further material or machines and that can be safely performed by patients suffering from unspecific CLBP. However, some limitations must be acknowledged. The observed local EIH effects were only measured immediately post exercise and no conclusions can be drawn regarding its temporal stability. Even though the PPT measurements were performed by an experienced single researcher, this researcher was not blinded and a possible experimenter bias must be acknowledged. Besides, this measurement is rater‐ as well as participant‐dependent as the pressure is applied manually and as this method is semi‐objective where the participant has to indicate when the applied experimental pressure becomes painful for the first time and a potential measurement bias must be acknowledged. A further potential limitation of PPT measurements might also be the risk of pain summation, as the time between consecutive PPT measurements was less than 20 s. Lastly, participants of this study were familiarised with the performed exercises in the initial meeting and exercises were subsequently performed under supervision ensuring proper technique. Yet, this supervision is of course not granted in an everyday setting and future studies should test the safety and feasibility of such exercise regiments in real‐world scenarios.

## 5 Conclusion

This study reveals that an easy‐to‐perform 10 min isometric core stabilisation exercise results in local lumbar EIH compared to a control session. Hence, this kind of exercise can be seen as clinically relevant as it acutely reduces local pain sensitivity at the lower back area between L1 and L5 of about 19%, while no additional equipment (e.g., weights or machines) is needed. Yet, no EIH effects are observed remotely hinting to the fact that primarily local pain inhibiting mechanisms are active by this exercise. Furthermore, patients with a higher degree of catastrophizing show a reduced hypoalgesic effect.

## Conflicts of Interest

The authors declare no conflicts of interest.

## Supporting information


Table S1.

## Data Availability

The data that support the findings of this study are available from the corresponding author upon reasonable request.
