# Supplementary material for: Ten Minutes of Core Stabilisation Exercise Result in Local Exercise‐Induced Hypoalgesia in Patients With Chronic Unspecific Low Back Pain
Source: Eur J Pain. 2025 Feb 8;29(3):e4794. doi: 10.1002/ejp.4794 (PMC11807238; doi:10.1002/ejp.4794)
Supplement: Supplementary file 1 — Table S1. [file EJP-29-0-s001.docx]

**Supplementary Material Table 1:** Individual pre and post PPT data measured at the different landmarks. * = p < 0,05; ** = p < 0,01; *** = p < 0,001 compared to pre of the respective group (Bonferroni adjusted post-hoc testing); ## = p < 0,01 compared to the core exercise group at the respective time point (Bonferroni adjusted post-hoc testing). Data presented as means ± SD (min – max). *ŋ^2^_p_ = partial eta-squared, PPT = pressure pain threshold, L = lumbar vertebrae.*

| **Variable** | **Group** | **Pre Mean ± SD (Min-Max)** | **Post Mean ± SD (Min-Max)** | **Relative change [%]**  **Mean ± SD**  **(Min-Max)** | **Main Effect**  **Time** | **Main Effect Intervention** | **Interaction Time x Intervention** |
| --- | --- | --- | --- | --- | --- | --- | --- |
| Local (pooled) | core-trainig | 56.6 ± 20.6 (14.8 - 92.2) | 67.5 ± 26.1 *** (17.1 – 124.3) | 18.8 ± 12.6  (-3.1 – 44.8) | F(1, 29) = 36.477  **p < 0.001** η²_p_ = 0.557 | F(1, 29) = 3.242  p = 0.082 η²_p_ = 0.101 | F(1, 29) = 31.823  **p < 0,001** η²_p_ = 0.523 |
|  | control | 58.5 ± 24.0 (18.6 – 113.6) | 58.4 ± 23.3 ^###^ (14.1 – 102.8) | 0.0 ± 8.8  (-24.6 – 17.6) |  |  |  |
| Remote (pooled) | core -trainig | 41.3 ± 12.5 (11.8 – 80.8) | 42.5 ± 13.6 (11.4 - 81.4) | 2.3 ± 9.9  (-20.5 – 18.8) | F(1, 29) = 0.001  p = 0.977 η²_p_ = 0.000 | F(1, 29) = 0.000  p = 0.995 η²_p_ = 0.000 | F(1, 29) = 6.845  **p = 0.014** η²_p_ = 0.191 |
|  | control | 42.5 ± 14.1 (12.3 – 72.1) | 41.3 ± 13.7 * (13.3 - 67.1) | -2.3 ± 7.1  (-17.3 – 20.1) |  |  |  |
| L 4/5 left | core -trainig | 56,6 ± 22,7 (14.6 - 102.5) | 67.1 ± 29.4 ***  (17.3 – 137.8) | 19.7 ± 22.1  (-24.0 – 60.3) | F(1, 29) = 10.268  **p = 0.003** η²_p_ = 0.261 | F(1, 29) = 1.485  p = 0.233 η²_p_ = 0.049 | F(1, 29) = 22.686  **p < 0 .001** η²_p_ = 0.439 |
|  | control | 59.1 ± 24.6  (18.5 - 122.6) | 59.0 ± 25.7 ^#^  (14.3 - 115.5) | -0.4 ± 11.0  (-22.7 – 23.5) |  |  |  |
| L 4/5 right | core -trainig | 55.2 ± 19.4  (12.8 - 92.5) | 57.3 ± 22.3 ***  (20.1 - 102.6) | 19.7 ± 17.9  (-20.9 – 60.0) | F(1, 29) = 19.045  **p < 0.001** η²_p_ = 0.396 | F(1, 29) = 1.452  p = 0.238 η²_p_ = 0.048 | F(1, 29) = 28.572  **p < 0.001** η²_p_ = 0.496 |
|  | control | 57.3 ± 22.3  (20.1 - 102.6) | 58.3 ± 24.4 ^##^  (15.5 - 108.4) | 1.2 ± 10.2  (-22.9 - 17.7) |  |  |  |
| L 3/4 left | core -trainig | 56.1 ± 21.6  (13.8 - 94.8) | 66.1 ± 26.9 ***  (15.8 - 128.3) | 18.5 ± 18.9  (-25.4 – 79.1) | F(1, 29) = 14.008  **p < 0.001** η²_p_ = 0.326 | F(1, 29) = 5.655  **p = 0.024** η²_p_ = 0.163 | F(1, 29) = 17.602  **p < 0.001** η²_p_ = 0.378 |
|  | control | 56.2 ± 23.5  (18.1 - 125.1) | 56.0 ± 22.5 ^###^  (12.5 - 107.5) | 0.5 ± 15.0  (-30.9 – 37.5) |  |  |  |
| L 3/4 right | core -trainig | 56.0 ± 21.5  (14.4 - 95.6) | 69.3 ± 28.3 ***  (17.3 - 134.5) | 24.8 ± 17.0  (-10.6 – 58.2) | F(1, 29) = 36.579  **p < 0.001** η²_p_ = 0.558 | F(1, 29) = 2.455  p = 0.128 η²_p_ = 0.078 | F(1, 29) = 26.429  **p < 0.001** η²_p_ = 0.477 |
|  | control | 59.0 ± 25.5  (15.9 - 123.7) | 57.6 ± 22.3 ^###^  (13.3 - 109.0) | -0.5 ± 12.3  (-24.6 – 27.9) |  |  |  |
| L 2/3 left | core -trainig | 55.0 ± 21.8  (16.0 - 95.4) | 67.7 ± 27.8 ***  (18.6 - 117.7) | 23.5 ± 23.7  (-9.8 – 90.1) | F(1, 29) = 14.663  **p < 0.001** η²_p_ = 0.336 | F(1, 29) = 1.203  p = 0.282 η²_p_ = 0.040 | F(1, 29) = 18.151  **p < 0.001** η²_p_ = 0.385 |
|  | control | 59.1 ± 26.6  (18.5 - 117.0) | 58.5 ± 24.5 ^###^  (15.2 - 110.3) | 1.1 ± 17.3  (-29.3 – 50.8) |  |  |  |
| L 2/3 right | core -trainig | 58.1 ± 22.2  (15.1 - 114.6) | 66.4 ± 25.7 **  (15.3 - 120.2) | 14.8 ± 19.6  (-29.0 – 71.9) | F(1, 29) = 7.492  **p < 0.010** η²_p_ = 0.205 | F(1, 29) = 2.650  p = 0.114 η²_p_ = 0.084 | F(1, 29) = 9.388  **p = 0.005** η²_p_ = 0.245 |
|  | control | 58.8 ± 24.3  (17.8 - 104.6) | 58.1 ± 24.1 ^##^  (13.4 - 102.6) | -1.0 ± 12.1  (-30.6 – 23.2) |  |  |  |
| L 1/2 left | core -trainig | 58.5 ± 23.2  (16.4 - 113.7) | 70.4 ± 28.1 ***  (18.2 - 114.0) | 19.9 ± 16.6  (-6.0 – 47.1) | F(1, 29) = 22.412  **p < 0.001** η²_p_ = 0.436 | F(1, 29) = 5.501  **p = 0.026** η²_p_ = 0.159 | F(1, 29) = 24.051  **p < 0.001** η²_p_ = 0.453 |
|  | control | 60.8 ± 27.5  (19.1 - 129.2) | 58.6 ± 24.0 ^###^  (14.8 - 101.6) | -2.1 ± 10.7  (-22.7 – 17.2) |  |  |  |
| L 1/2 right | core -trainig | 57.2 ± 22.9  (15.6 - 105.0) | 66.9 ± 29.1 ***  (18.8 - 123.8) | 17.0 ± 20.9  (-43.9 . 62-3) | F(1, 29) = 23.343  **p < 0.001** η²_p_ = 0.446 | F(1, 29) = 0.312  p = 0.581 η²_p_ = 0.011 | F(1, 29) = 6.359  **p = 0.017** η²_p_ = 0.180 |
|  | control | 60.2 ± 27.3  (19.5 - 131.6) | 61.0 ± 25.5  (13.4 - 113.2) | 3.0 ± 17.1  (-31.3 – 58.9) |  |  |  |
| thumb pad | core -trainig | 46.0 ± 13.8  (12.2 - 80.3) | 47.4 ± 15.5  (10.7 - 78.8) | 2.4 ± 15.0  (-26.3 – 36.6) | F(1, 29) = 0.334  p = 0.568 η²_p_ = 0.011 | F(1, 29) = 0.007  p = 0.934  η²_p_ = 0.007 | F(1, 29) = 2.023  p = 0.166 η²_p_ = 0.065 |
|  | control | 47.1 ± 15.8  (12.9 - 75.3) | 46.6 ± 16.3  (14.8 - 74.6) | -0.5 ± 10.4  (-16.3 – 26.8) |  |  |  |
| forehead | core -trainig | 36.6 ± 14.7  (11.3 - 102.5) | 37.6 ± 14.6  (12.0 - 98.7) | 3.0 ± 10.2  (-26.1 – 26.6) | F(1, 29) = 0.422  p = 0.521 η²_p_ = 0.014 | F(1, 29) = 0.012  p = 0.912 η²_p_ = 0.000 | F(1, 29) = 5.918  **p = 0.021** η²_p_ = 0.169 |
|  | control | 37.9 ± 14.9  (11.6 - 90.1) | 36.0 ± 11.7  (11.8 - 59.6) | -3.2 ± 10.0  (-36.7 – 18.4) |  |  |  |
